# Vaccinating wild badgers against bovine tuberculosis: an overview of 16 years of vaccine delivery in England

**DOI:** 10.3389/fvets.2026.1901413

**Published:** 2026-08-27

**Authors:** Andrew Robertson, Clare H. Benton

**Affiliations:** 1 Department for Environment, Food and Rural Affairs, Bristol, United Kingdom; 2 Animal and Plant Health Agency, National Wildlife Management Centre, Gloucestershire, United Kingdom

**Keywords:** badger, BCG, bovine tuberculosis, *Mycobacterium bovis*, vaccination, wildlife disease

## Abstract

**Introduction:**

The European badger (*Meles meles*) is wildlife host for Mycobacterium bovis (*bovine tuberculosis*) in the UK and several European countries, complicating efforts to control the disease in livestock. Badger vaccination using the injectable BadgerBCG vaccine has been available in England since 2010 as a non-lethal tool to reduce TB transmission in badger populations and reduce risks to cattle. Despite long-standing interest, national deployment has historically been limited, and questions remain about operational feasibility.

**Methods:**

Here we provide a comprehensive overview of 16 years of badger vaccination delivery in England, summarizing national deployment trends, geographical expansion, recorded welfare-related observations, operational efficiency in government led programmes, and the effects of culling history on trapping success.

**Results:**

From 2010 to 25 there were 84,085 trap nights, resulting in 25,692 vaccine doses administered to wild badgers in England. Visual welfare assessments by lay vaccinators recorded >99% of badgers as normal for demeanor, respiration, condition, and movement. Minor injuries such as abrasions to the forelimbs were recorded in 4.2% of badgers with serious injuries extremely rare (0.03% of captures) and veterinary callouts required in only 0.04% of events. Trapping efficiency varied by month and trap location and was significantly higher in unculled populations, than those where culling had recently taken place. In post-cull areas, trapping success increased steadily with time, which may suggest partial population recovery and increasing trap encounter rates.

**Discussion:**

These results show that badger vaccination in England has expanded substantially and can be delivered across diverse landscapes, including areas recently subject to culling. While vaccination using traps is unlikely to become a landscape-wide tool due to costs and logistical constraints, it can act as a targeted intervention in areas where badgers contribute meaningfully to disease transmission. These findings provide robust operational evidence to inform policy and support the strategic deployment of badger vaccination as part of England's evolving bTB control strategy.

## Introduction

Bovine tuberculosis (bTB) is a globally significant zoonotic disease that infects a wide range of species, with serious implications for both animal and human health. In numerous regions, efforts to control bTB are hindered by the presence of wildlife hosts, which may act as ongoing sources of infection for livestock and other susceptible species (1).

In the UK and Ireland the European badger (*Meles meles*) is the principal wildlife host for *Mycobacterium bovis*, although the pathogen is also present in other wild species (2–4). Efforts to reduce disease transmission between badgers and cattle have focused primarily on badger culling, which seeks to reduce opportunities for transmission by lowering badger population densityIn England, a large-scale Badger Control Policy, combining badger culling with additional cattle control measures has been in place since 2013, with evidence that this package of measures is associated with reductions in TB incidence in cattle (5, 6).

The use of vaccines is an alternative, non-lethal approach to controlling disease in wildlife populations. Although challenging to deliver, there are number of examples where wildlife vaccination has been successful at controlling diseases which threaten wild populations themselves (7), or pose a risk to humans or livestock (8). The Bacille Calmette Guerin, or BCG vaccine is the primary vaccine for the control of bTB, and it has been demonstrated as safe and effective in a range of both wild and domesticated species (9). The BadgerBCG vaccine has been licensed for use in England since 2010, underpinned by evidence of its safety and efficacy in badgers (10, 11). Large scale badger vaccination is also taking place in Ireland. Like culling, vaccination seeks to reduce transmission of infection within and between host populations. However, it does so by providing protection against infection and disease progression rather than by reducing host abundance.

Laboratory trials have demonstrated that BadgerBCG reduces disease severity and progression of TB in badgers under high-challenge conditions (12, 13). Field trials in England and Ireland have also provided evidence of vaccine protection against natural challenge, with vaccine efficacy varying from approximately 50%−80% depending on diagnostic methods used (13–15). Importantly, vaccination of adult badgers has been associated with reduced TB incidence in unvaccinated cubs, consistent with herd immunity effects (14). Population-level modeling suggests that, despite incomplete protection and limited coverage, vaccination has the potential to reduce TB prevalence in both badgers and cattle (16–18). Supporting this, field evidence from long-term studies shows declines in TB prevalence in vaccinated badger populations (19, 20). Large scale badger vaccination delivery following culling in Ireland has also shown that badger vaccination is associated with disease outcomes in cattle which are comparable to those from continued badger culling in some situations (21). However, to date, no large-scale field trials have been conducted in England to assess the impact of badger vaccination on cattle TB incidence and this remains a key evidence gap, and a likely contributing factor to reported low levels of confidence in badger vaccination among farmers (22, 23).

In England, BadgerBCG is administered via intramuscular injection to badgers captured in cage traps, after which they are given a temporary mark and released. Given the protected status of badgers, trapping badgers for the purpose of vaccination in England requires licensing from Department for Environment Food and Rural Affairs (Defra) and Natural England, and can only be conducted by trained personnel acting under veterinary direction.

In 2021, the UK government announced a phased end to badger culling in England, with a planned expansion of badger vaccination (24). There remains considerable skepticism among farmers that it is possible to trap and vaccinate badgers at scale, particularly in culled populations (25), however early analyses of field data from government-led badger vaccination projects in post-cull areas showed encouraging results (26). Against this backdrop, there is a clear need for a comprehensive assessment of vaccination delivery to date. Using national datasets covering all licensed badger vaccination activity between 2010 and 2025, the objectives of this study were to: (i) quantify long-term trends in vaccine deployment and geographic expansion; (ii) summarize observations recorded during routine lay visual welfare assessments; (iii) assess operational performance and trapping efficiency within government-led vaccination programmes; and (iv) investigate the influence of culling history on trapping success, including temporal changes in trapping efficiency following the cessation of culling. We also present updated cost estimates for vaccination delivery and discuss the implications of these findings for the strategic use of badger vaccination within England's evolving bovine tuberculosis control programme.

## Methods

Badger vaccination in England is carried out by capturing free-living badgers in cage traps placed at locations showing evidence of badger activity, including setts (burrow systems), runs, and latrines. Once in place, traps are pre-baited for several days so that badgers become accustomed to entering them.

**Figure 1:**
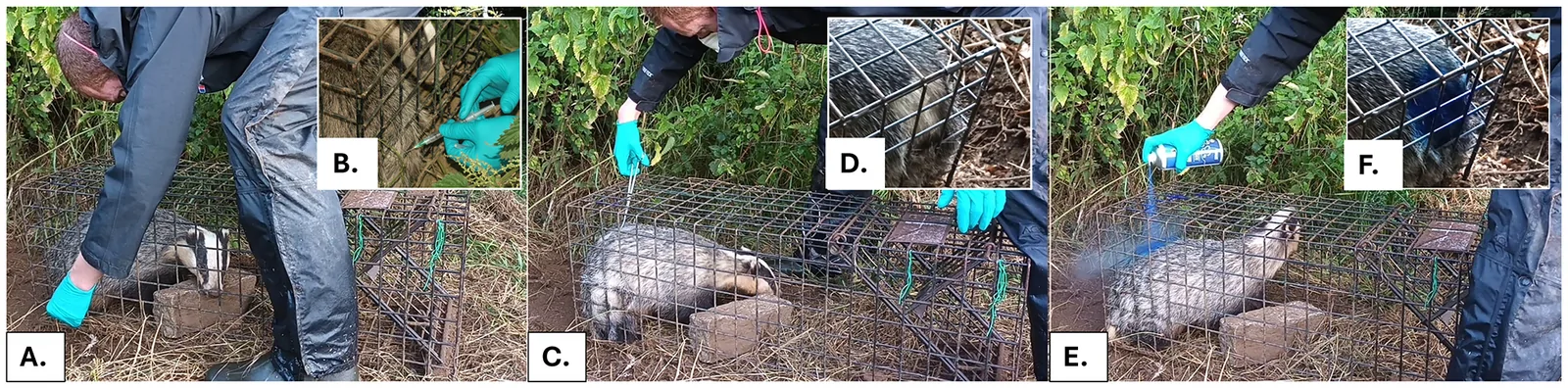
Stages of the badger vaccination process; vaccine administration by intramuscular injection to the thigh muscle **(A**, **B)**, fur clip mark using scissors to remove the outer guard hairs **(C, D)** and stock marker **(E, F)**. The stone in the trap is part of the trigger mechanism.

Traps are then typically set to catch for two consecutive nights (although longer trapping periods may occasionally be used) and checked early the following morning. Consequently, a trapping location may generate captures on multiple consecutive mornings, including both newly captured and previously marked badgers. All captured badgers are given a visual welfare assessment before being vaccinated via intramuscular injection, temporarily marked using a fur clip and stock marker, and then released (Figure 1). Recaptured badgers [identified by temporary fur clips and stock marker (27)], receive a lay visual fitness to release check and are released without re-vaccination (Figure 1). Permanent individual marking is not routinely undertaken during vaccination operations because these activities are conducted for disease-control purposes and permanent marking would require anesthesia and further veterinary involvement. Temporary marking provides sufficient identification of recently vaccinated animals during trapping operations whilst minimizing handling time and avoiding additional marking procedures (27).

### Veterinary oversight

Badger vaccination operations are conducted under veterinary oversight. As vaccination by intramuscular injection constitutes an act of veterinary surgery, lay vaccinators operate under a specific exemption to the Veterinary Surgeons Act (1966), subject to defined training requirements and veterinary direction. A prescribing veterinary surgeon is responsible for authorizing vaccine supply and providing operational direction, including agreed protocols for animal handling and welfare management. Lay vaccinators carry out routine visual fitness and condition checks and record observations, but do not diagnose conditions or make treatment decisions. Where observations indicate a potential welfare concern, a veterinary surgeon is consulted, and all decisions regarding veterinary examination, the use of anesthesia, attendance at site, or euthanasia are made by the veterinary surgeon. In accordance with license conditions, a veterinary surgeon is available on call throughout vaccination operations.

The data described here relating to fitness, condition and injury status are derived from routine lay visual checks conducted to determine fitness for vaccination and fitness for release. These records do not constitute a comprehensive welfare or ethical assessment of trapping and vaccination, and they cannot capture the full welfare impact of the process. Accordingly, the analyses presented are limited to describing the nature and frequency of observations recorded during these routine checks, rather than assessing overall welfare impact.

### Data collection and management

Badger vaccination in England is licensed by Defra and Natural England and licensees are required to submit data detailing trapping activities and outcomes. From 2010 to 2021 information relating to badger vaccination trapping operations were recorded in paper forms by vaccinators and then submitted to the licensing authority. Since 2022, badger vaccination activities have also been recorded using the TRAPP smartphone app developed by the World Veterinary Service (WVS), which is now becoming the primary method of reporting.

Precise point locations (in the form of 10 figure OS grid references) of all traps are recorded, along with the location type; badger sett; or remote location (e.g., a badger run or latrine away from the footprint of the sett). On vaccination mornings the trap outcomes are recorded (badger, not-tripped, tripped but empty, non-target species), along with the badger age (adult or cub) and whether badgers were vaccinated or if they were recaptures (as indicated by the presence of a temporary fur clip and stock marker). Five parameters are recorded during routine lay visual fitness and condition checks; demeanor; respiration; body condition; injuries and movement, with badgers recorded as either normal, common departure or abnormal against these criteria. If common departures are noted, such as minor injuries or poor body condition, the specific details are recorded. In 2025 the parameters were renamed to alert, breathing, condition, injuries and movement as part of a review of operational procedures. Although terminology changed, the underlying assessment criteria remained broadly consistent throughout the study period. For consistency, the 2025 data was converted back into the previous parameter names so that the data could be combined and summarized more easily.

For the current study, a single combined dataset was compiled containing all available records of licensed badger vaccination activity in England from 2010 to 2025. This dataset includes all badgers captured and vaccinated under Defra and Natural England licenses for the purpose of managing *M. bovis* infection in wildlife. Some scientific research projects have licenses from Defra/Natural England in addition to licenses issued under the Animals (Scientific Procedures) Act (ASPA). Data from these projects was included in the study, but data from research projects licensed under APSA only were not available. The dataset includes licensed badger vaccination activity from across England, spanning multiple geographic regions and TB risk areas, including both areas with and without recent histories of badger culling. Vaccination was undertaken in a range of landscapes including intensive agricultural areas, mixed farmland, conservation land and low-risk TB areas where vaccination has increasingly been used as a response to localized outbreaks of infection.

Within the national licensing dataset, contextual information on delivery models and costs was obtained from APHA to deliver badger vaccination across a range of geographic scales, with substantial investment since 2021 in vaccinating previously culled areas. The Vaccinating East Sussex Badgers (VESBA) project is a farmer-led vaccination programme operating within East Sussex. Established in 2021 and delivered under the direction of Cliffe Veterinary Group, VESBA aims to vaccinate badgers across approximately 250 km^2^ and represents the largest coordinated industry-led badger vaccination initiative undertaken in England. Information from APHA and VESBA was used to provide illustrative examples of alternative delivery models and to summarize operational cost estimates.

### Trapping efficiency during government-led vaccination

All statistical analyses were conducted in R (28). To investigate differences in trapping efficiency between unculled and post-cull populations under similar conditions, the dataset was restricted to Animal and Plant Health Agency (APHA) delivered vaccination operations from 2022 to 2025, yielding 21,426 trapping records. Each record represented an instance of a single trap being set on one date.

A generalized linear mixed model (GLMM) was fitted using the lme4 package (29), with a binomial response describing whether each trap captured a badger (1) or not (0). Fixed effects included month, trapping type (trapping at the badger sett or trapping remotely away from the sett at another area of badger activity e.g., a run or latrine) and population status (culled vs. unculled area, defined at the operational area level due to limited spatial precision in available culling records). An interaction term between month and trapping type was included to test whether differences in trapping efficiency between sett and remote traps varied through the vaccination season due to temporal changes in badger activity and patterns of habitat use.

Calendar year (2022–2025) and trapper ID were incorporated as random effects to account for temporal variation and operator-specific differences.

### Post-cull trapping recovery analysis

To assess how trapping efficiency changes with time since culling ceased, the APHA dataset was further restricted to post-cull areas only (*n* = 12,358 trapping records). The GLMM included fixed effects for month, trapping type (sett or remote as above) and years since the cessation of culling (1–4 years). An interaction between trapping type and years post-cull was included to test whether recovery in trapping efficiency following the cessation of culling differed between traps located at setts and those deployed remotely at other signs of badger activity. Calendar year and trapper ID were again treated as random effects.

### Lay visual fitness and condition assessments during badger vaccination operations

All complete records from badger vaccination operations since 2010 from both government (APHA and its predecessors) and non-government delivered badger vaccination (hereafter referred to as NGO) were used to produce a summary table of recorded welfare-related observations from routine lay visual fitness and condition checks. These data are based on a visual assessment by a trained lay vaccinator of a conscious trapped badger in cage traps and do not represent a veterinary opinion or diagnosis.

## Results

### Badger vaccination delivery 2010–2025

From 2010 to 2025 there were a total of 84,085 trapping events (instances where a trap was set to catch) as part of badger vaccination activities in England, resulting in 31,679 instances where a badger was caught and 25,693 instances where a badger was vaccinated. While badgers are given a temporary mark to indicate they have been vaccinated, they are not permanently uniquely marked, so it is not possible to ascertain the total number of different individuals caught or vaccinated. In total there were 18,583 vaccines administered to adult badgers, 6,789 to badger cubs and 321 to badgers of unknown age (i.e., not clearly a cub or adult).

Trap day (e.g., first, second or subsequent trapping morning) is not routinely recorded within vaccination records. Traps are typically operated for two consecutive nights at a given location, although longer trapping periods are occasionally used. Therefore, trapping day was inferred using vaccination date and location information, with the earliest recorded trapping date at a given location in a given year classified as the first trapping morning and all later dates classified as subsequent trapping mornings. Of the 25,597 instances where a badger was vaccinated with location information (point location or site name; 96 vaccination records lacked sufficient location information and were excluded from this analysis), 19,740 (77%) were trapped and vaccinated on the first morning of trapping, with the remaining 5,857 (23%) vaccinated on later mornings. A total of 5,957 recaptured badgers with noticeable marks (fur clip and stock marker) were captured during vaccination operations. The majority of these (*n* = 5,350) were caught on the second or subsequent morning, such that recaptured badgers accounted for 48% of badgers captured on these mornings. A small number of recaptured badgers were also caught on the first morning of trapping at a given location (*n* = 607 or 3% of first morning captures), likely indicating movement from other previously vaccinated locations.

**Figure 2:**
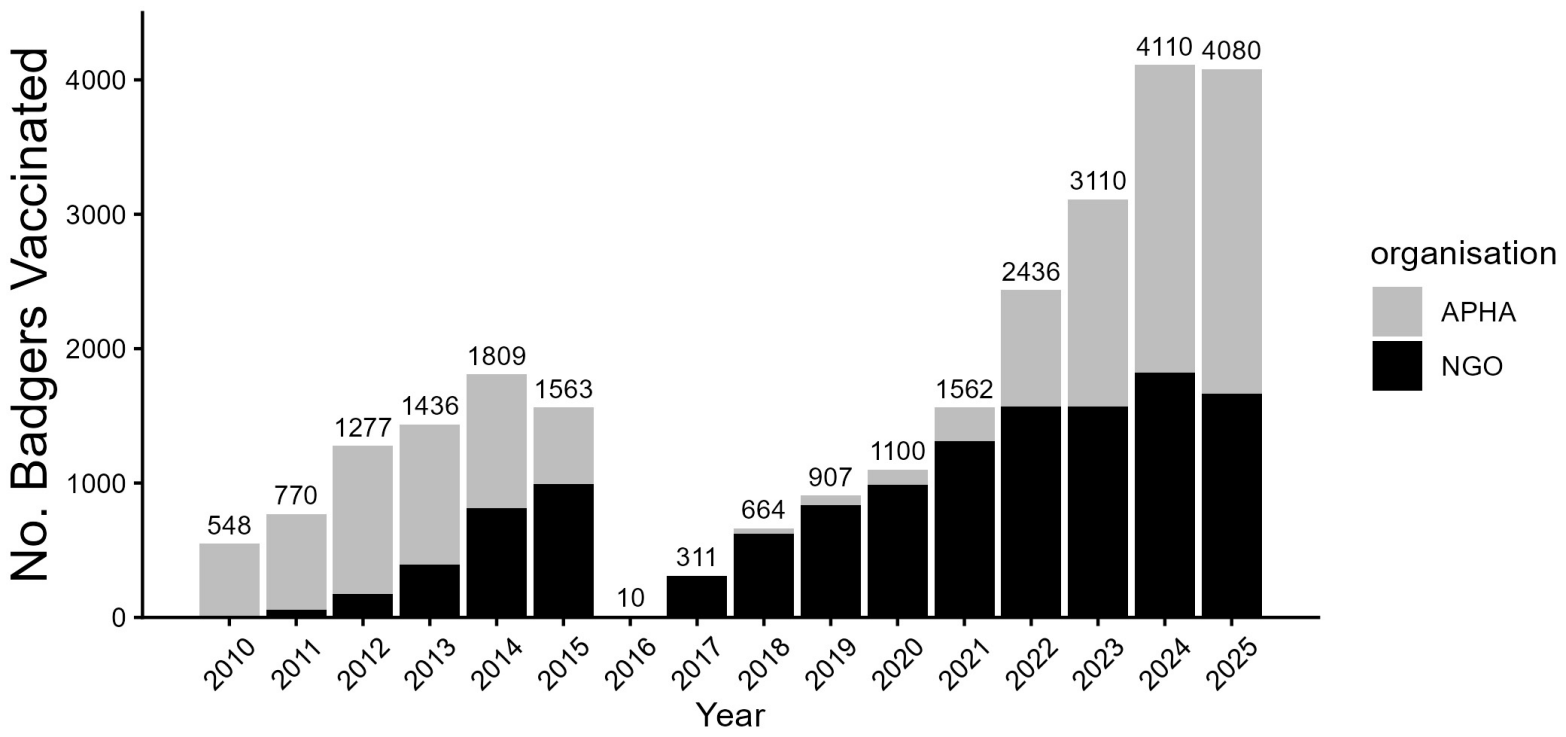
Number of doses of BadgerBCG delivered to trapped badgers from the period 2010–2025. Bars are divided by organization; Animal Plant Health Agency (APHA) and Non-government Organizations (NGO).

Annual vaccination numbers increased steadily from 2010 to 2015 before falling to 10 vaccinations in 2016 due to the global BCG shortage (Figure 2). Since this low point, the number of badgers vaccinated annually in England has increased significantly, peaking in 2024, with 4,110 badgers vaccinated, which is the highest number vaccinated in England to date (Figure 2). The number then fell slightly in 2025 to 4,080 badgers.

**Figure 3:**
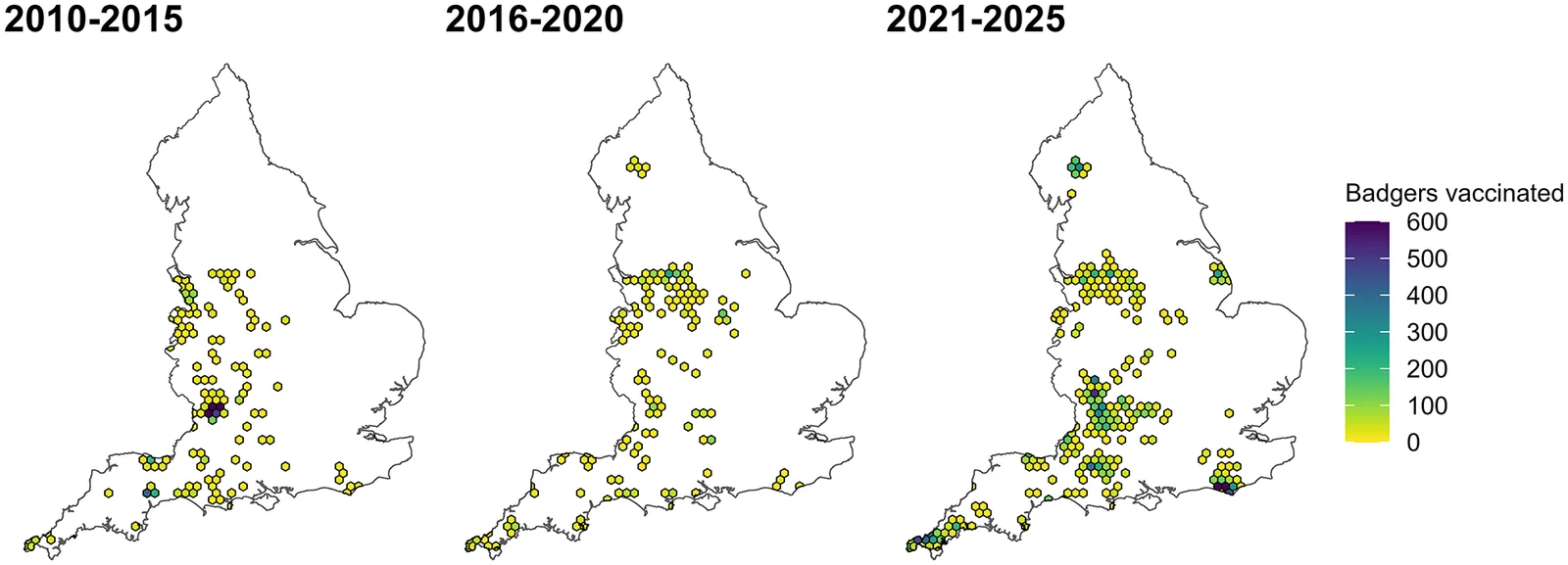
Geographical extent of BadgerBCG deployment over time. Data represent doses of vaccine delivered within 10 km hexagon cells, rather than number of individuals as badgers are not permanently marked and may be revaccinated across years.

Early deployment of badger vaccination was driven largely by government projects delivered by APHA, with steady growth in NGO delivered vaccination over the period from 2010 to 2015. The NGOs involved comprise of a mixture of organizations and groups largely from the wildlife and conservation sector. During the years 2017–2022 the majority of badgers were vaccinated by NGO groups, but recent growth since then has been achieved in part through a large increased vaccination by APHA from 2022 to 2025, along with further NGO delivery through a Defra funded industry led scheme in East Sussex (Figure 3).

Alongside a growth in number of badgers vaccinated, the geographic spread of delivery vaccination has also increased over time (Figure 3). In 2025, badgers were vaccinated in 21 counties across England and there are now several large contiguous vaccination areas (Figure 3). Vaccination is taking place in former badger control areas, and in areas with unculled badger populations. In recent years badger vaccination has also increasingly being used in Low-Risk TB Areas in Northern England.

### Lay visual fitness and condition assessments during badger vaccination operations

The available data represented 31,543 badger capture events during vaccination operations between 2010 and 2025. A small number of capture records lacked complete data for one or more parameters (*n* = 135, < 0.5%), with the number missing varying slightly between parameters. For four of the five parameters recorded during routine lay visual fitness and condition checks (demeanor, respiration, body condition and movement), badgers were classified as normal in more than 99% of cases (Table 1). These records describe observations made during routine lay visual fitness and condition assessments at the time of capture. Observed abnormalities represented a mixture of trapping-related findings along with pre-existing conditions.

**Table 1:** Lay visual fitness and condition assessment data for trapped badgers recorded against five parameters.

Parameter	Normal		Abnormal		Common departure		Examples of common departures
	*n*	%	*n*	%	*n*	%	
Demeanor	31,490	99.8	1	0.003	49	0.2	Shivering, aggressive or lethargic
Respiration	31,497	99.9	1	0.003	43	0.1	Panting or fast breathing rate
Body condition	31,327	99.3	0	0	215	0.7	Thin or poor condition, hair loss, some injuries (old wounds, cataracts etc.)
Injuries	30,216	95.8	7	0.022	1,320	4.2	Abrasions on forelimbs/head, damaged claws, bite wounds, existing eye conditions (e.g., cataracts) or old scars
Movement	31,524	100.0	1	0.003	14	0.0	Limping, shivering

The number of instances and percentage of total are recorded against “Normal,” “Common Departure” from normality and “Abnormal.”

Common departures from normality (worthy of note and record) were rare, but slightly higher for the injuries parameter at 4.2% (Table 1). The majority of common departure injuries were abrasions or grazes to the nose or forelimbs (70%) or bite wounds to the head/rump, or other scars and old injuries (20% of injuries). Other injuries recorded referred to badger eyes (3.9%—often cataracts, evidence of existing blindness or minor cuts surrounding the eye area) and broken claws (2%). Injuries to the mouth area were recorded in 13 instances (1% of injuries recorded) including evidence of blood or broken teeth. Evidence of abscesses or swelling to the face were recorded in 10 instances (0.8% of injuries) prior to vaccination. However, there were no recorded instances of swelling or other reactions post-vaccination in any vaccinated badgers over the 16-year period.

Abnormal fitness or condition observations of badgers were noted in 10 cases, which equated to 0.03% of trapped badgers (Table 1). Abnormal cases included a badger which was drooling and shaking prior to vaccination (demeanor) and a recaptured badger with its jaw caught in the trap mesh (movement). There were seven badgers with serious injuries which were classed as abnormal, this included four cases with injuries to the face such as lacerations or puncture wounds and one individual with a large abscess on one side of the face. Veterinary examination indicated that these injuries were not recent and were therefore considered unlikely to be associated with the trapping event. One injured badger was recorded with broken teeth, and another injured badger was recorded with a swollen leg. The badger with broken teeth was not identified during the welfare assessment, but during blood sampling as part of a research project. One badger was also found dead in the cage, but with no clear signs of injury (recorded as abnormal respiration). This badger had not been vaccinated. One badger also died while under anesthesia, again as part of blood sampling unrelated to the vaccination process. The tenth abnormal record related to a badger with its jaw caught on the trap mesh (movement parameter) which was then freed by the vaccinator with no apparent resulting injuries.

There were 12 recorded instances where a vet was contacted for advice by a lay vaccinator following the visual fitness and condition assessment (0.04% of capture events). This included the first nine instances described above, along with three further instances where badgers were observed with common departures during the assessment and additional veterinary advice was sought. Of the 12 instances where veterinary advice was sought, vets decided a field visit was required for further examination in seven instances. In one further instance, a vet was also called out to assess an injured pheasant which had been caught in the trap. Badgers were vaccinated and released in 9/12 instances (75%) where veterinary advice was sought. Of the three badgers not released, one was found dead in the cage (as described above), and another died during an anesthetic procedure undertaken in response to an exceptional handling situation.

The third badger was found with a large laceration on the side of the muzzle, under anesthesia the wound was inspected which highlighted a serious injury to the nose/rhinarium and a decision was taken by the vet to euthanise the badger on welfare grounds.

### Overview of government-led vaccination (APHA)

**Figure 4:**
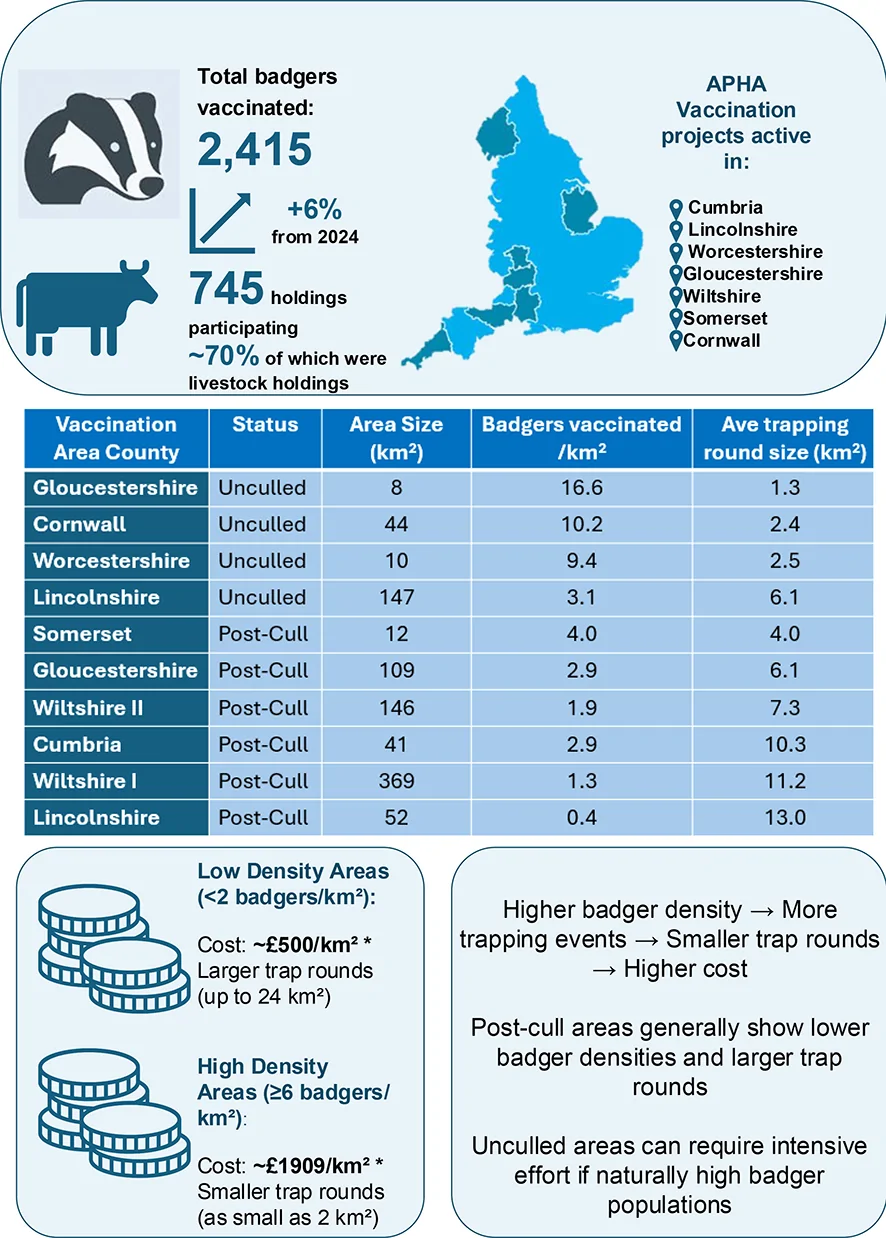
Government led vaccination in 2025

The year 2025 was the peak delivery year to date with APHA vaccinating a total of 2,415 badgers in England; an increase of 6% from the previous highest year in 2024. In 2025, APHA delivered badger vaccination across 11 areas spanning seven counties: Cornwall, Wiltshire, Cumbria, Gloucestershire, Lincolnshire, Somerset and Worcestershire, of which six areas had previously been subject to industry-led culling. Within the vaccination areas a total of 745 holdings were included, an estimated 70% of which were livestock holdings. The remainder of the holdings represented a range of land types such as nature reserves, council owned land, rural land without livestock, National Trust properties (Figure 4).

Variation in underlying badger density, both due to natural variation in the baseline population between different areas and land class types and as a result of previous management interventions, has a strong influence on the effort required to effectively trap and vaccinate the population. This has an obvious impact on the cost to vaccinate (as measured in terms of cost per km^2^).

The estimated cost per square kilometer of APHA-delivered badger vaccination in 2023 (defined as the direct costs of delivery including staff time during vaccination rounds, provision of local on-call veterinary cover and vaccination consumables, but excluding travel-related costs) varied between approximately £500 per km^2^ in areas of low badger density (< 2 badgers per km^2^) and £1,909 per km^2^ in areas of higher badger density (>6 badgers per km^2^). However, because a greater number of badgers are captured and vaccinated in higher-density populations, the estimated cost per vaccine dose administered showed the opposite pattern, averaging approximately £166 per badger vaccinated in high-density populations compared with £579 per badger vaccinated in low-density populations.

### Overview of an industry-led vaccination project (VESBA—Vaccinating East Sussex Badgers)

Between 2021 and 2025 the VESBA project delivered a total of 2,855 doses of BadgerBCG to wild badgers using a total of 14 lay vaccinators recruited from the local community. The project started with a 55 km^2^ area, delivering 78 vaccine doses in 2021. This increased rapidly to 250 km^2^ and 481 doses in 2022. By 2025 the project area covered approximately 265 km^2^, delivering 878 vaccine doses per year. The project was funded by Defra, costing a total of £2.27 m over the 5 year period. Delivery costs (excluding project management and communications costs—so broadly equivalent to government costs above) were £1,445 per km^2^, or £549 per badger vaccinated.

#### Trapping efficiency during government-led badger vaccination operations

**Figure 5:**
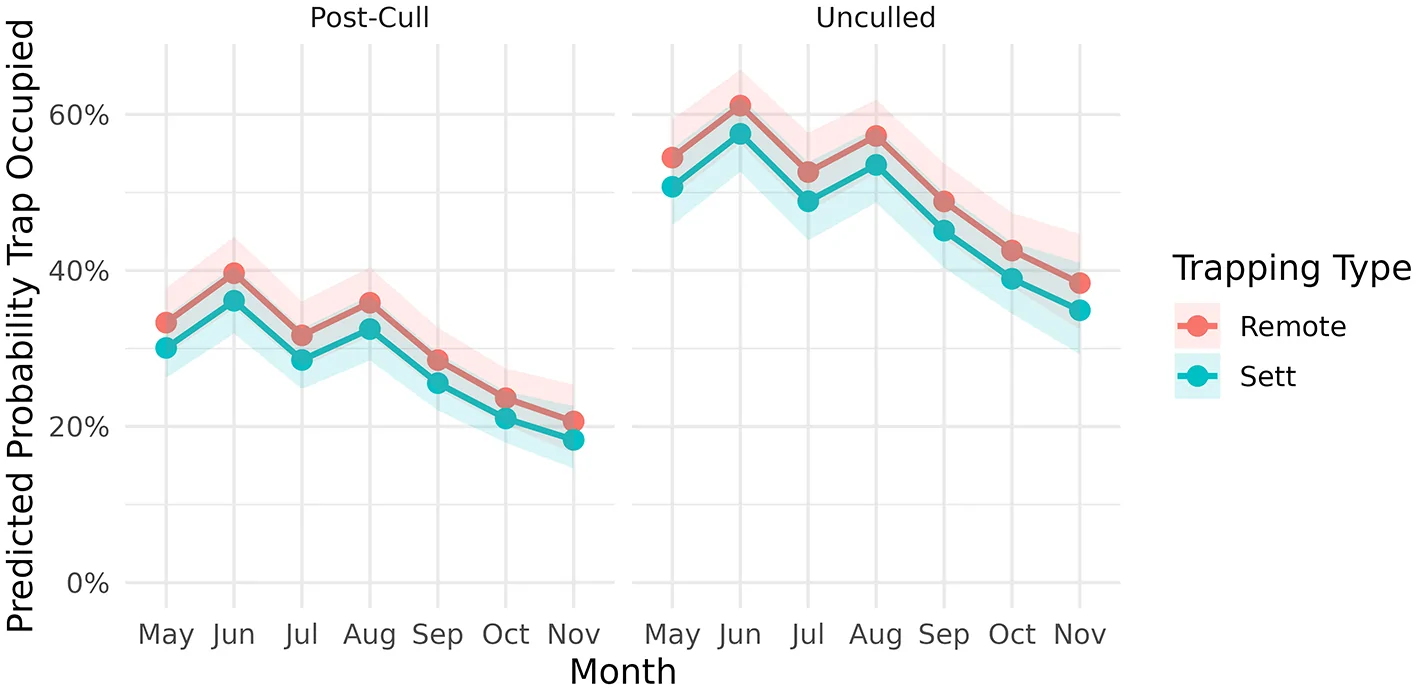
Model predicted variation in trapping efficiency during APHA delivered badger vaccination operations based on data collected 2022–2025.

The probability of a trap successfully catching a badger varied between months ($\chi_{\left(5,11\right)}^{2}$ = 255, *p* < 0.001), with trapping efficiency falling slightly toward the end of the season and also with trapping type (sett or remote) as shown in Figure 5 ($\chi_{\left(10,11\right)}^{2}$ = 23, *p* < 0.001) with trapping efficiency consistently slightly higher in remote traps as compared to trapping on sett. Marginal means, averaged over month and population type, indicated higher occupancy rates for remote traps (40%) than for traps placed at setts (36%). Trapping efficiency also varied significantly between post-cull and unculled populations with significantly higher trapping efficiency in unculled badger populations as compared to trapping in post-cull settings ($\chi_{\left(10,11\right)}^{2}$ = 476, *p* < 0.001). Marginal means, averaged over month and trapping type, indicated higher predicted occupancy rates in unculled areas (49%) when compared to post-cull areas (29%). There was no significant interaction between population status and trapping type (interaction term $\chi_{\left(11,12\right)}^{2}$ = 0.008, *p* > 0.05).

**Figure 6:**
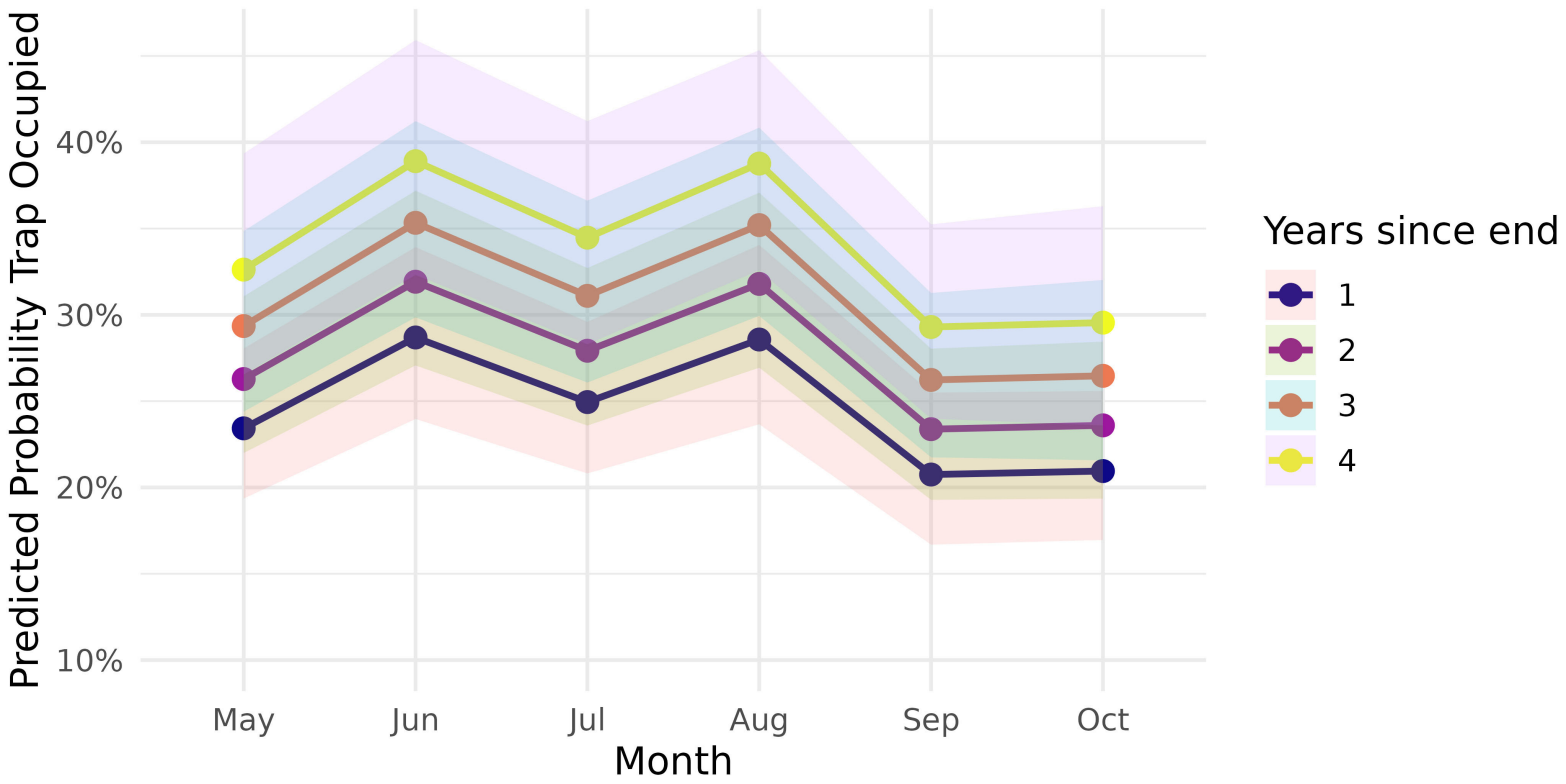
Model predicted variation in trapping efficiency during APHA delivered badger vaccination operations in post-cull areas based on data collected 2022–2025.

When the dataset was censored to only include records from APHA-delivered badger vaccination in post-cull operations, there was no significant interaction between years since the cull had ended (min = 1, max = 4) and trapping type (remote or on sett trapping; interaction term $\chi_{\left(11,12\right)}^{2}$ = 0.7, *p* > 0.05). As in the full model, trapping efficiency was slightly higher at remote traps than at setts; however, in this case the difference was non-significant ($\chi_{\left(11,12\right)}^{2}$ = 3.2, *p* = 0.08). Trapping efficiency varied significantly by month as in the full model above, falling slightly toward the end of the season (Figure 6; $\chi_{\left(11,12\right)}^{2}$ = 61.5, *p* < 0.001). Trapping efficiency increased significantly with time since culling ceased ($\chi_{\left(11,12\right)}^{2}$ = 12.5, *p* < 0.001). Marginal means of predicted trap occupancy, averaged over month, rose from 25% in the first year to 34% 4 years after culling ceased. However, trapping efficiency remained below that observed in unculled populations (49%), indicating only partial recovery rather than convergence.

## Discussion

Badger vaccination has been available as a tool for controlling TB in badgers since 2010 however, its rollout in England has been constrained by several barriers, including low farmer confidence in badger vaccination (22, 23), alongside the requirements for training and licensing, and the logistical challenges associated with cage trapping badgers. The data presented here show that, although badger vaccination remained relatively small-scale and localized for much of the past decade, deployment has expanded substantially in recent years. Vaccination is now being delivered across large areas of England by both government and non-government organizations, with the East Sussex pilot further demonstrating that industry-led vaccination at scale is feasible. Access to land is a critical prerequisite for delivering badger vaccination at scale. Previous social science research has identified persistent uncertainty among some farmers regarding both the effectiveness and practicality of badger vaccination as a tool for controlling bovine tuberculosis (22, 30). Although land-access outcomes are routinely considered during operational planning, records have not been collected consistently across all projects and years in a form that would allow robust national estimates of participation or refusal rates to be derived from the dataset analyzed here. Nevertheless, experiences from projects such as VESBA where land access was granted for >90% of the area demonstrate that strong local engagement, veterinary leadership and collaboration with farmers and landowners can facilitate large-scale delivery. Continued efforts to communicate operational experience, scientific evidence and local disease-control objectives are likely to be important in maintaining and expanding participation in future vaccination programmes.

When carrying out large scale wildlife management it is important that the animal welfare implications of methods such as trapping are properly monitored and assessed (31). Although the welfare data collected as part of badger vaccination is based on visual inspection only, this has resulted in a large data set presented here of over 30,000 captures. Encouragingly, badgers were recorded as “normal” with no injuries or abnormal movement, behavior, demeanor or respiration in the vast majority of cases. Veterinary advice was only sought by vaccinators in a handful of instances over the 16-year study period, with only three instances involving the death of a badger. This suggests that an increase in vaccination delivery nationally is unlikely to have marked negative impacts on badger populations, or require significant veterinary resource to treat injured badgers as a result of the vaccination process. Crucially, there was no evidence of adverse reactions to the vaccine itself, at least within the relatively short time (several minutes) that badgers were observed post-vaccination. This supports previous conclusion that BCG is a safe vaccine in badgers and a range of other species (9, 32). Common departures in the form of minor injuries were the most common welfare observation (4.2% of badgers) in the form of abrasions on the forelimbs, nose and head, likely from badgers attempting to escape once caught. Close inspection of carcasses from badgers cage trapped during previous government culling programs in England show a broadly similar pattern with no injuries in the majority of cases (33), but with a higher rate than those recorded here (12% of 5,964 culled badgers). Close examination of culled badgers during the same study also revealed injuries to the jaws and mouth which may not be detected in a visual welfare assessment. We note that the previous study (33) included data from badgers trapped using older designs of cage traps which have subsequently been modified to reduce rates of traps injuries (specifically the coating of all traps with a polymer to reduce abrasions and a modification to the door mechanism to reduce tooth and jaw injuries). Nevertheless, despite the small number of reported injuries to the mouth and teeth in the current study (*n* = 13), it is possible that injures such as these are under reported. It is also important to recognize that the observations reported here represent conditions identified during routine visual assessments and do not necessarily all indicate injuries caused by trapping or vaccination. Some abnormalities recorded during vaccination operations were judged to be pre-existing conditions unrelated to the trapping event.

Although the cage traps used in badger vaccination meet internationally recognized humane trapping standards, debate remains regarding the adequacy of these welfare thresholds (34). Whilst the low percentages of reported welfare concerns from the vaccination trapping data presented here are encouraging, it is important to recognize the likely under-reporting in this data, due both to the self-reported nature of the data and the limited opportunity for close observation of trapped badgers. The observed injury rate of 4.2% should be interpreted as a minimum estimate. Previous post-mortem examination of cage-trapped badgers reported injury rates of approximately 12%, including oral and dental injuries that may not be readily detectable during routine visual assessment of conscious animals. Consequently, some injuries are unlikely to be detected by the field-based assessments used here and may require further studies specifically aimed at investigating the welfare impacts of trapping badgers. Setting aside physical injuries (as these are only one dimension of welfare), the process of trapping badgers itself does impose a welfare cost on badgers (35) and this always needs to be balanced with the potential benefits of any intervention.

The recent expansion of government delivered vaccination, also demonstrates that despite the challenges of trapping in culled badger populations (26), it is feasible to deliver vaccination within former badger control areas, even when vaccination follows culling in the subsequent season. This counters negative perceptions among some farmers who believe that large- scale vaccination, particularly within culled populations is not practical (30). These projects also highlight the huge variation in badger density within potential target areas for badger vaccination and how this impacts logistics and cost (see Figure 4).

The logistical challenges and costs associated with live trapping have prompted a long-standing interest in oral vaccination as a potential alternative delivery route. Experimental studies in have demonstrated that oral administration of BCG can provide protection against tuberculosis in badgers (20), and a range of vaccine baits have been developed and trialed on captive and wild badgers (36). However, studies have struggled to demonstrate consistent efficacy from vaccine baits (37). While no oral vaccine work is ongoing in England, research elsewhere using alternative vaccines may offer future avenues for oral vaccine development (38).

Our analyses demonstrate that whilst trapping efficiencies within government-delivered vaccination programmes are consistently high, trapping efficiencies are, as expected, lower in post-cull populations. Trapping rates were 49% in unculled areas, and 29% in previously culled areas. These are similar if not slightly higher than published rates of trapping efficiency for previous government delivered badger culls which were 20%-30% on the first night of trapping at given locations (39). An important limitation is that cull status was defined at area level rather than the individual trap level. While all traps classified as post-cull were located within licensed culling areas, information was not available on whether the specific land parcel containing each trap was accessible to culling, nor whether any badgers had been removed from that immediate location. As a result, some traps may have been situated in areas where local badger density was less affected by culling than elsewhere within the same control area. This would tend to reduce the apparent difference between post-cull and unculled populations, suggesting that the estimated 49 vs. 29% contrast in trapping efficiency is likely to be conservative. In unculled populations, relatively stable social structures, higher baseline densities and clearer field signs (which form the basis of where traps are placed) are all likely to contribute to greater trap success (26). Data from post-cull areas show that trapping efficiency increased progressively with time since culling ceased. This pattern is consistent with population recovery and increasing trap encounter rates following the cessation of culling, although the relative contribution of ecological and operational factors could not be determined from the current dataset. The slightly higher trapping efficiency observed at remote locations is likely a consequence of targeted deployment at signs of recent activity, whereas sett trapping typically involves placing multiple traps to ensure coverage of all entrances, including those used less frequently. This difference reflects deployment strategy rather than the intrinsic superiority of remote trapping. Mixed deployment strategies targeting both sets and remote locations are standard in badger vaccination programmes and reflect established field practice. Trapping efficiency also varied with month, with a gradual decline toward the end of the vaccination season. This seasonal pattern is consistent with established understanding of badger ecology and behavior, and is likely driven by a combination of changes in ranging activity, food availability and bait attractiveness as the season progresses (40). Later in the summer and early autumn, increased availability of natural food resources may reduce trap visitation rates, while changes in weather conditions and vegetation cover can further influence trap encounter probability. These findings reinforce existing guidance on the timing of trapping operations and highlight the importance of accounting for seasonal variation when planning vaccination effort and interpreting trapping success.

Although these analyses are based on data from England, the operational insights generated here, particularly around vaccination delivery at scale, trapping efficiency, seasonal effects and post-intervention population recovery are likely to be relevant to other wildlife vaccination and disease-control programmes internationally that rely on live capture and field delivery under veterinary oversight. Live-capture vaccination methods analogous to those described here have been applied internationally, for example through trap–vaccinate–release programmes used to control rabies in carnivores where oral vaccination is impractical (7, 41). These programmes share common challenges around trapping strategies highlighting the wider relevance of the operational insights presented in this study.

This study represents the first national-scale synthesis of badger vaccination delivery in England. By combining operational, welfare and trapping-efficiency data from more than 25,000 vaccine administrations over a 16-year period, the study provides an evidence base against which future vaccination programmes can be assessed and highlights the practical opportunities and constraints associated with large-scale deployment. While the numbers displayed here indicate a sizeable increase in badger vaccination, it should be noted that the numbers involved are still significantly below annual numbers of badgers culled in England in recent years. For example, in England approximately 20,000–40,000 badgers were culled annually from 2020 to 24. England is not alone in its aspirations to increase badger vaccination delivery. Badger vaccination has significantly increased in recent years in the Republic of Ireland and is now a key part of their bTB eradication strategy (42), although a different trapping method (stop restraints) are used for badger capture. Badger vaccination in the ROI is government funded, which is also the case for most projects in England.

Although evidence from experimental studies, field studies and modeling suggests that badger vaccination can reduce infection and disease progression in badger populations (11), a key evidence gap remains its impact on bovine tuberculosis incidence in cattle under field conditions in England However, evidence from the Republic of Ireland indicates that replacing continued badger culling with vaccination was associated with cattle herd TB outcomes that were not inferior to those observed under continued culling in most circumstances (21). As badger vaccination expands across England, there is an opportunity to address this evidence gap through carefully designed field studies and analysis of cattle TB data. Approaches such as Before-After-Control-Impact (BACI) designs, combined with routine cattle surveillance, whole-genome sequencing and wildlife epidemiological investigations, could help quantify the contribution of vaccination to disease control and identify the circumstances under which vaccination delivers the greatest benefit to cattle.

Although badger vaccination is now being delivered at a greater scale than at any point since its introduction, its relatively high operational costs means it is unlikely to be suitable as a landscape-wide intervention. In England, bovine TB herd breakdowns result in significant economic losses, with mean costs estimated at £23,000 per affected herd, although this varies with markedly herd type and size (43). The cost-benefit of badger vaccination is likely to vary by location, depending on the costs of programme delivery (which are influenced by badger density), the rate and economic impact of cattle herd TB breakdowns, and the effectiveness of vaccination in reducing disease transmission to cattle. Future work is needed to examine the interplay between these factors and how they influence the financial viability of implementing badger vaccination at scale.

Logically vaccination is likely to offer the greatest value when deployed strategically in areas where evidence suggests that badgers contribute significantly to transmission of TB to cattle. Whole genome sequencing (WGS) studies increasingly show that the role of badgers in local transmission dynamics is highly variable across space, with some areas showing strong evidence of badger–cattle transmission and others dominated by cattle-to-cattle spread (44–46). This spatial heterogeneity suggests that a one size fits all approach to wildlife intervention is unlikely to deliver consistent benefits. A more effective and proportionate approach is to improve our understanding of the drivers of infection in each problem area, using tools such as cluster identification, source attribution and integrated epidemiological investigation, and to design evidence-based packages of measures across both wildlife and cattle accordingly. Within such a framework, badger vaccination remains an important option, particularly where it can help reduce infection pressure from badger populations to local cattle, and its principal value is likely to lie in targeted deployment where local evidence suggests it can deliver the greatest benefit.

## Data Availability

The datasets presented in this article are not readily available because the data in the study relates to licensed wildlife management operations and within the data there are specific trap locations and vaccinator (personal) data which are sensitive and cannot be shared. Requests to access the datasets should be directed to andrew.robertson1@defra.gov.uk.
